# Gaming control using a wearable and wireless EEG-based brain-computer interface device with novel dry foam-based sensors

**DOI:** 10.1186/1743-0003-9-5

**Published:** 2012-01-28

**Authors:** Lun-De Liao, Chi-Yu Chen, I-Jan Wang, Sheng-Fu Chen, Shih-Yu Li, Bo-Wei Chen, Jyh-Yeong Chang, Chin-Teng Lin

**Affiliations:** 1 Department of Electrical Engineering, National Chiao Tung University, Hsinchu 300, Taiwan; 2 Department of Computer Science, National Chiao Tung University, Hsinchu 300, Taiwan; 3 Brain Research Center, National Chiao Tung University, Hsinchu 300, Taiwan; 4 Division of Medical Engineering Research, National Health Research Institutes, Miaoli 350, Taiwan

**Keywords:** Electroencephalography (EEG), Brain-computer interface, Dry EEG sensor, Cognitive applications

## Abstract

A brain-computer interface (BCI) is a communication system that can help users interact with the outside environment by translating brain signals into machine commands. The use of electroencephalographic (EEG) signals has become the most common approach for a BCI because of their usability and strong reliability. Many EEG-based BCI devices have been developed with traditional wet- or micro-electro-mechanical-system (MEMS)-type EEG sensors. However, those traditional sensors have uncomfortable disadvantage and require conductive gel and skin preparation on the part of the user. Therefore, acquiring the EEG signals in a comfortable and convenient manner is an important factor that should be incorporated into a novel BCI device. In the present study, a wearable, wireless and portable EEG-based BCI device with dry foam-based EEG sensors was developed and was demonstrated using a gaming control application. The dry EEG sensors operated without conductive gel; however, they were able to provide good conductivity and were able to acquire EEG signals effectively by adapting to irregular skin surfaces and by maintaining proper skin-sensor impedance on the forehead site. We have also demonstrated a real-time cognitive stage detection application of gaming control using the proposed portable device. The results of the present study indicate that using this portable EEG-based BCI device to conveniently and effectively control the outside world provides an approach for researching rehabilitation engineering.

## Introduction

The monitoring of brain activity is widely used for investigative neuroscience and rehabilitation engineering [1]. The brain-computer interface (BCI) technique has become a major tool that provides a direct communication pathway between the brain and the external world by translating signals from brain activities into machine codes or commands [2-5]. The acquisition of brain activities by BCIs can be divided into two different categories [4]: invasive BCIs [6,7] and noninvasive BCIs [8,9]. An invasive BCI is implanted directly into the grey matter of the brain to obtain the highest quality of brain activity signals or to send external signals into the brain [7]. However, invasive BCIs depend on surgical techniques and are potentially risky because of the interaction between the device and brain tissues when used in the long term. Therefore, noninvasive BCIs have become another major BCI research direction. These noninvasive devices are worn on the outside of the head and are removable. Recently, electroencephalogram (EEG)-based BCIs have been shown to provide a feasible and noninvasive method to communicate between the human brain and external devices [10,11]. The use of EEG signals has become the most common approach for BCIs because of their usability and strong reliability [12,13]. In recent years, the advanced designs of the sensors and system techniques have made it possible to integrate the sensors into portable acquisition devices to measure a wide variety of physiological signals. The development of EEG-based BCIs and their corresponding applications have also been reported [14-16]. A BCI system that is based on steady-state visual-evoked potentials (SSVEPs) has been commonly used for controlling functional neuroprostheses [8,17-19]. Gollee *et al*. used a BCI system that was based on SSVEPs combined with a functional electrical stimulation (FES) system to allow the user to control stimulation settings and parameters [17]. In addition, a P300-based BCI has also been developed for disabled users [10,20-22]. The current applications of P300-based BCI systems range from controlling a virtual hand [10] to neuroprostheses [21,23]. EEG-based BCIs provide a reliable, fast, and efficient solution for the communication between humans and computers. However, most of the above-mentioned BCIs focus on feasible applications by using general systems or sensors. Measuring EEG signals with a portable BCI device in a comfortable manner during daily life is still an important issue that requires further study [15].

The most frequently used wet- or micro-electro-mechanical-system (MEMS)-type EEG sensors for EEG-based BCI devices have some limitations [24], such as skin abrasion and the required use of conductive gel; moreover, they are time-consuming, uncomfortable, and often painful for users [25-27]. These sensors are also inappropriate for long-term EEG measurements because the EEG signal quality may degrade over an extended period of time because of skin regeneration and/or the drying of the conductive gel [24,28]. In addition, most of the non-gel-based dry electrodes were made using the MEMS technique [25,26,29,30]. However, the dry MEMS electrode technique relies on invasive penetration into the skin to acquire the EEG signals [25,26]. In addition to the drawback of skin penetration, MEMS electrodes are also more costly to manufacture than gel-based or other types of dry electrodes. Our recent study utilized dry foam-based electrodes to acquire forehead EEG signals without any skin preparation or gels [31]. However, the size of most of the EEG-based BCI devices is too large for them to be considered a portable device [32], which is inconvenient for users. Therefore, developing a portable EEG-based BCI device of a smaller size (smaller than 5 × 5 × 5 cm^3^) with zero-preparation, dry EEG sensors is an important goal.

In this study, we developed a wearable, EEG-based BCI device with a novel dry foam-based sensor and demonstrated a cognitive application of gaming control. This device consisted of a wireless EEG acquisition device and a computer. The wireless EEG acquisition device included dry foam sensors and a wireless EEG acquisition module. The proposed dry foam sensors worked without the application of conductive gel; however, they were able to provide good conductivity to effectively acquire an EEG signal. Moreover, this sensor can be properly integrated into the wireless EEG acquisition device. In contrast to other portable BCI devices using the wet sensors, which require a skin preparation process [32,33], users using the proposed device can monitor their EEG states more quickly, comfortably and effectively during daily life and can transmit EEG signals to a personal computer to process signals directly. In addition, a real-time focusing detection algorithm [34] was implemented in our device as an EEG-based gaming interface to detect the real-time cognitive state of the user in a comfortable manner. The use of this device complements other existing BCI approaches for investigating the cognitive states of neuronal activation and behavioral responses in daily life.

## Materials and methods

### A. Design of the dry EEG sensors

**Figure 1:**
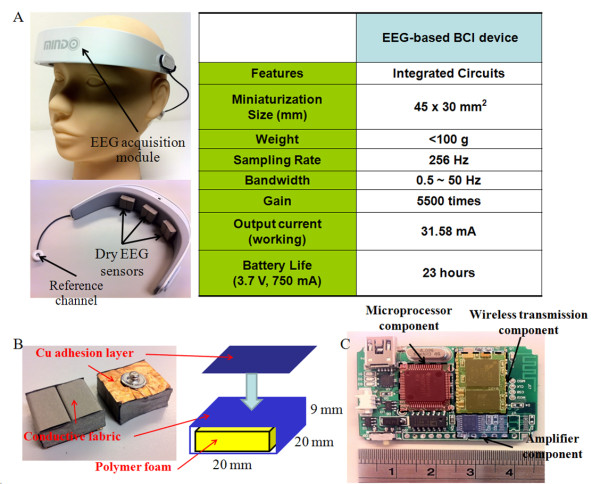
**(A) The proposed wearable EEG acquisition device and the dry EEG sensors with their performance characteristics**. (B) A magnified view of the proposed dry foam-based EEG sensor. (C) A schematic diagram of the circuit board of the wireless EEG acquisition device.

The proposed dry foam-based EEG sensor was specific designed to contact the skin of the forehead with the use of a conductive polymer foam made of a urethane material with a compression set of about 5~10%, as shown in Figures 1A and 1B. The conductive foam was covered with a 0.2-mm-thick taffeta material that was made from an electrically conductive polymer fabric (conductivity of about 0.07 ohm/cm^2^) and was coated with Ni/Cu on all of its surfaces to establish an electrical contact that was similar to that of silver EEG sensors. A 0.2-mm layer of Cu was used as an adhesion layer that was then connected to the wireless EEG acquisition module. The proposed dry foam EEG sensors were 20 × 20 × 9 mm^3^. The corresponding design specifications and the equivalent circuit of the skin-sensor interface for dry EEG sensors can be found in our previous study [35].

### B. Wireless EEG acquisition module

**Figure 2:**
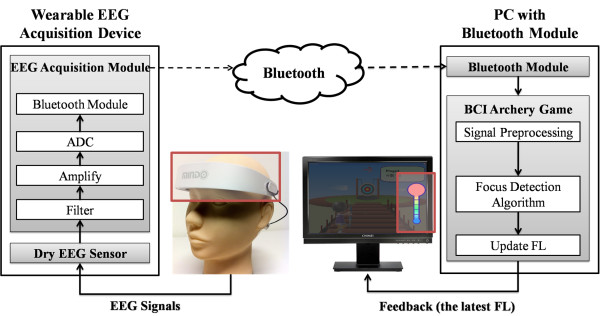
**This schematic shows the proposed wearable/wireless EEG-based BCI device and its application to gaming control**.

Figure 1C and Figure 2 show the wireless EEG acquisition module and its application to gaming control. It was used to acquire EEG signals from the dry EEG sensors and included the (INA2126, Texas Instruments, USA), an acquisition component (AD8609, Analog Devices, USA), a microprocessor component (MSP430, Texas Instruments, USA), and a wireless transmission component (BM0403, Unigrand Ltd., Taiwan) [36]. To amplify and filter the EEG signals, a pre-amplifier, a band-pass filter (0.5~50 Hz) and an analog-to-digital converter (ADC) were embedded into our circuit board as a bio-signal amplifier and acquisition component modules. The gain of the amplifier and acquisition component was set to approximately 5500. An ADC with 12-bit resolution was used to digitize the EEG signals, with a sampling rate of 256 Hz for the amplified and filtered EEG signals. In the microprocessor component, the EEG signals that were probed using an ADC were digitally stored. A moving average filter with the frequency at 60 Hz was then applied to reject any power-line interference before the wireless transmission. A Bluetooth module, BM0403 (Unigrand Ltd., Taiwan), was included in the wireless transmission portion of the circuit. It is important to note that the module was fully compliant with the specifications for a Bluetooth v2.0+ EDR and a Printed Circuit Board (PCB) antenna. In total, the size of the proposed wireless EEG acquisition module was approximately 4.5 × 3 × 0.6 cm^3^, and we were able to embed this module into the mechanism of our wearable EEG-based BCI device. This module was operated at 31.58 mA with a 3.7-V DC power supply. Most important, this module was able to operate continuously for 23 hours using a commercial 750 mAh Li-ion battery.

### C. The mechanism of the wearable EEG-based BCI device

The quick-placement mechanism for the proposed EEG-based BCI device was designed to let the dry EEG sensors attach to the user's forehead (F10) easily and quickly, as shown in Figure 1A. This device consists of three dry foam sensors and a wireless EEG acquisition module that contains a battery. An elastic band was adjustable to fit the users' head sizes, as indicated in Figure 1A. This mechanism was also used to maximize the skin-sensor contact area to maintain low impedance while probing the EEG signals using the dry EEG sensors [37]. This mechanism did not lead to any permanent or detrimental effects to the forehead skin. Noted that all of the channels of the porposed device are both used the dry foam-based electrodes. The application of the wearable EEG acquisition device allowed users to monitor their EEG signals more conveniently and comfortably.

### D. Gaming control via users' focus levels measured by EEG signals with the proposed device

**Figure 3:**
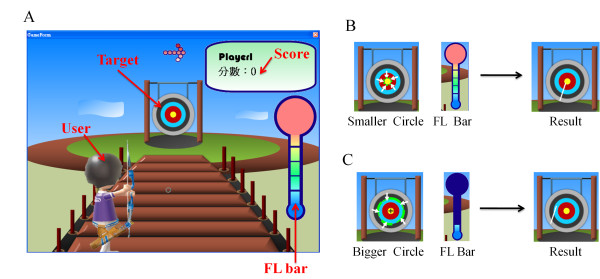
**(A) The interface for the EEG-based BCI archery game**. The visualized gaming results (FL values) for higher and lower FL values are shown in (B) and (C), respectively.

To demonstrate the performance of the proposed EEG-based BCI device with dry sensors in daily life applications, we proposed a computer game controlled by users via the mental focusing feature from the EEG signals. The interface of this game is shown in Figures 3. All of the users who played this archery game equipped themselves with the proposed EEG-based BCI device. The users had to make a shot; they then obtained a score based on the distance between the arrow on the target and the center of the target. There was a bar on the right of the screen, a target at the center of the screen, and a score at the top right of the screen (Figure 3A). The bar indicated the focusing level (FL) of this user during the gaming (Figure 3B and 3C). In other words, the FL value was the main controller of the game. If the value of the FL was high, then the shot was close to the center of the target, and then the gaming score was high. If the value of the FL was low, the shot was far from the center of the target and resulted in a lower score. The user's task was to make the FL value as high as possible by firing the shot close to the center of the target. Users had 10 s to complete one shot, and the total score was calculated after ten shots.

**Figure 4:**
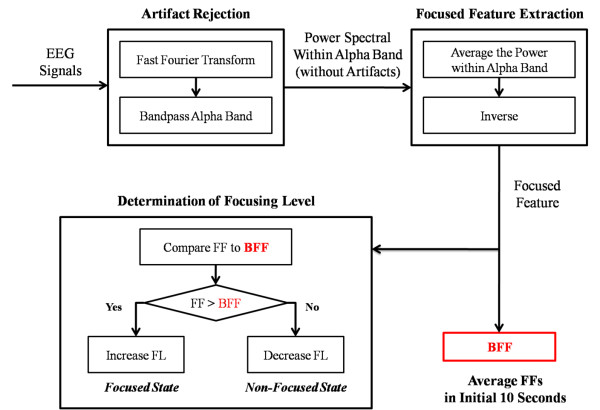
**Flowchart of the FL detection algorithm**.

To measure the FL values of the users, a simple, real-time, mental focusing level detection algorithm for gaming control was proposed. The flowchart of this FL detection algorithm is shown in Figure 4. The FL detection algorithm includes three major steps: 1) rejection of the artifact signals, 2) extraction of the focusing feature and 3) determination of the FL values. First, preprocessing of the original EEG signals was performed to reject the noise signals [1]. It is well known that the mentally focused state is highly associated with the alpha rhythm (8~12 Hz) of an EEG in the forehead region [1,36,38], and the noise artifacts were located in frequency regions that were different from the alpha rhythm frequency range [1,36]. Accordingly, to reject the artifacts, a fast Fourier Transform was performed to obtain the EEG power spectrum patterns of the signals, and signals within the alpha band were retained.

Secondly, extraction of the focus feature was performed on the power spectrum within the alpha band. Previous studies [36,38] have shown that the power of the alpha rhythm of an EEG grows as the user's mental state changes from focused to unfocused cognitive states. Therefore, the alpha band is the main frequency band that we used to indicate the user's focused state in the present study [36,38], and the 8~12 Hz frequency band of the original EEG signals was selected for the FL detection algorithm. The Focus Feature (FF) is defined as the inverse of the average power in the alpha rhythm, as shown in equations (1-3):

(1) $$\begin{array}{ccc} X & =\left[\begin{array}{cccccc} X_{1} & X_{2} & X_{3} & .. & X_{511} & X_{512} \end{array}\right] & \\ Y & =\left[\begin{array}{cccccc} Y_{1} & Y_{2} & Y_{3} & .. & Y_{255} & Y_{256} \end{array}\right]\\ Y & =FFT\left(X\right) \end{array}$$

(2) $$P_{\alpha }=\frac{1}{5}\overset{12}{\underset{n=8}{\sum }}Y_{n}$$

(3) $$FF=PR_{\alpha }=1/P_{\alpha }$$

*X *indicates the recorded samples in 2-s, where *X*_n _is the nth sample. *Y *is the power spectrum of *X*, which is calculated by the FFT; *Y*_n _indicates the power in the nth rhythm. The average power within the alpha band *P_α _*is obtained by averaging the value of *Y *in the range from 8 to 12 Hz. *PR_α _*is the inverse of this average power in the alpha rhythm. The FF value is assumed to be equal to *PR_α_*. The power of the alpha rhythm has a negative relationship with the value of the FF. If the user is not focused, the power of the alpha rhythm will increase, and the value of the FF will decrease.

Lastly, a comparison of the user's current FF value with that at baseline was used to confirm whether or not the user was in a focused state and then to determine the FL based on the user's focused state. We assumed based on user feedback that the user was in a focused state in the beginning (baseline) and defined the user's FF at baseline as the baseline FF (BFF), which is the average of the FFs within the initial ten seconds. After we determined the BFF, the FF values were calculated every 2 s and were compared to the BFF. If the current FF value was higher than the BFF value, the user was considered to be in the focused state. If the current FF value was lower than the BFF value, the user was considered to be in the unfocused state. Finally, the values of the FL variation were determined according to the user's mental focus state. If the user was focused, the FL increased and vice-versa.

To apply this algorithm in our game, the gaming process consisted of ten trials, and each trial persisted for ten seconds, during which a shot was executed. The BFF was calculated during the initial ten seconds, and then the game began. For every shot, the FL was initialized to zero and increased or decreased according to the FF value. The FF values were calculated every 2 s and were then compared to the BFF. If the FF value was higher than the BFF during that 2 s, the FL increased by one level. If not, the FL decreased by one level. When the user pushed the mouse button, a circle on the target indicated the focus zone based on the user's FL level. This circle indicated the possible deviation of the shot from the center of the target and was scaled relative to the FL. If the FL was high, the circle became small, indicating that the possible deviation of the shot would be small and that the arrow would be close to the center of the target, and vice versa. Users attempted to focus during the game to make the FL as high as possible and to get a high score. After each shot, the score was calculated as the deviation of the shot from the center of the target and was summed to the user's total score, which was shown on the screen (Figure 3A). After ten trials, the total score was the sum of the ten scores from the ten shots. Noted that the users in all of the experiments performed the task without any pre-training or practice

### E. Verification of the FL algorithm with the proposed EEG device and dry sensors: comparison of the users' focused mental state with the FL algorithm

To confirm that the FL algorithm represented the user's level of focus, we compared the FL algorithm to a general measurement method for the focused mental state. According to the previous studies on mental focus, the most commonly used method for measuring the state of mental focus is called the "short-term memory test" [39-41]. In the beginning of this test, the user watches a rapid series of pictures over a few seconds. Next, a picture is shown to the user and the user must indicate whether or not this picture had been shown before. Previous authors indicated that the accuracy of this test is high when the user is in the focused state and low when the user is in the unfocused state [40]. Belojevic *et al*. confirmed that the accuracy of this test was high when users take the test in silence, indicating that the users were more focused, while the accuracy of the test was low when conducted under noisy conditions, indicating that the users were in an unfocused state [40].

**Figure 5:**
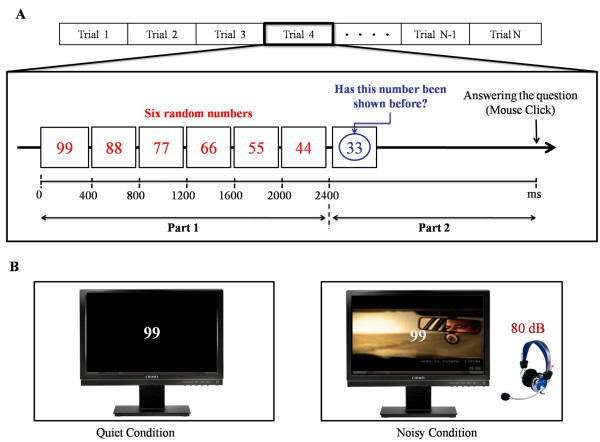
**(A) Schematic representation of the proposed short-term memory experiment**. This short-term memory test includes several trials, and each trial consists of two parts: 1) six numbers are presented to the user sequentially, and each number lasts for 400 ms; and 2) a number is presented to the user and the user must indicate whether or not the number had been shown before by using a mouse click. The total time of this short-term memory test was about 3 min, and the trial was repeated until the end of the testing period. (B) The experimental setup for the short-term memory test under the quiet and noisy conditions. Under the quiet condition, users were asked to take the test without any interruptions or noises. Under the noisy condition, users were asked to take the test with a randomly selected movie playing in the background on the screen and a sound playing via earphone. The sound consisted of a set of names spoken by a female voice at a random pace at 80 dB.

Following the above studies, we also designed a short-term memory experiment to ensure the validity of the FL algorithm with the proposed EEG device, as shown in Figure 5A. Users were asked to take the test under quiet and noisy conditions, as shown in Figure 5B. This short-term memory test included several trials, and each trial consisted of two parts: 1) six numbers were presented to the user sequentially, and each number lasted for 400 ms; and 2) a number was presented to the user and the user had to indicate whether or not the number had been shown before by using a mouse click, as shown in Figure 5A[39,40]. The total time for this short-term memory test was about 3 min, and the trial was repeated until the end of testing. Under quiet conditions, users were asked to take the test without any interruptions or noise. On the other hand, under the noisy condition, users were asked to take the test with a randomly selected movie played as a background on the screen and with sound played via earphone (Figure 5B) [42,43]. The sound consisted of a set of names, including that of the user, spoken by a female at a random pace at 80 dB [40,44]. Finally, the average accuracy of all the trials was calculated, and this value was used as an indicator to determine the user's level of focus.

Ten users participated in this short-term memory experiment, and all of them were right-handed and aged 24-27. All experiments took place during the afternoon with a computer and earphones, and users were asked to sit comfortably in front of the computer without crossing their legs [40,41]. After all of the short-term memory trials, then we also asked users to perform measurement experiment of the FF values and play the archery game with the proposed BCI device under quiet and noisy conditions. Finally, a *t*-test was performed on the FF values, the user focus levels (results from the short-term memory experiment) and the scores from the archery game to establish the relationship between these variables [40,41].

## Results and Discussion

### A. Characteristics of the proposed wearable EEG-based BCI device

**Figure 6:**
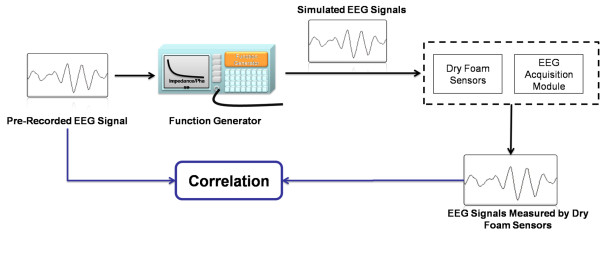
**Illustration of the pre-test experiment for the verification of the signal quality of the proposed dry sensor**.

**Figure 7:**
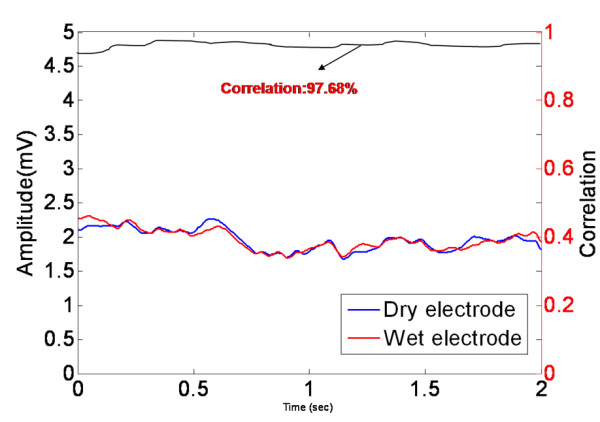
**Comparison of the pre-recorded EEG signals and the signals that were recorded using the dry EEG sensor**. The correlation value indicates the clarity of the signals that were measured using the dry EEG sensor.

In this subsection, we report the testing results of the dry sensors and the circuits to ensure that they are reliable for measuring EEG signals in daily life. The major components of the wearable EEG-based BCI device included the dry EEG sensors and their corresponding readout circuit. The dry EEG sensor was experimentally characterized with respect to the signal quality and the impedance between the skin-sensor interfaces. The pretest experiment that was used to verify the signal quality is shown in Figure 6. The aim of this pretest experiment was to identify any distortion that was caused by our dry EEG sensor during the EEG measurement. First, the EEG data were prerecorded using standard EEG sensors with conductive gel and were stored in a computer. Next, the EEG data were fed into a programmable function generator and were passed through a voltage divider to generate simulated human EEG signals. The simulated EEG signals were then fed into the dry EEG sensor and were compared with the recorded and the pre-recorded EEG data. Figure 7 shows the prerecorded EEG signals and the recorded signals from our proposed dry EEG sensor. The prerecorded EEG signals and the signals that were obtained using the dry EEG sensor were highly correlated at a level of 97.68%. This high level of correlation between the prerecorded EEG signals and the data obtained using the dry EEG sensor confirmed the clarity of the EEG signals that were recorded using the dry foam-based sensor.

**Figure 8:**
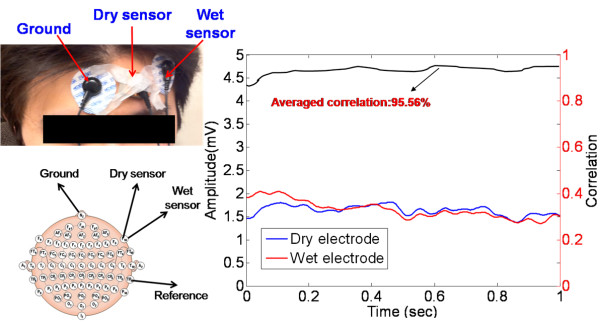
**Measurement of the EEG signals on the forehead site (F10) using the wet sensors and the proposed dry sensors**. The EEG signal correlation between these two sensors is shown in the right panel (95.56%).

Next, the correlation between the conventional wet EEG sensor and the dry EEG sensor was investigated. Figure 8 shows the sensor placements and the results of the EEG measurement after using dry/conventional EEG sensor pairs on the foreheads of the users (F10). The correlations between the signals that were obtained using the dry EEG sensor and the conventional wet EEG sensor were typically in excess of 95.56% for the forehead. Therefore, the performance of the EEG signal measurement using the dry foam-based EEG sensor was identical to that of the conventional wet EEG sensors.

**Figure 9:**
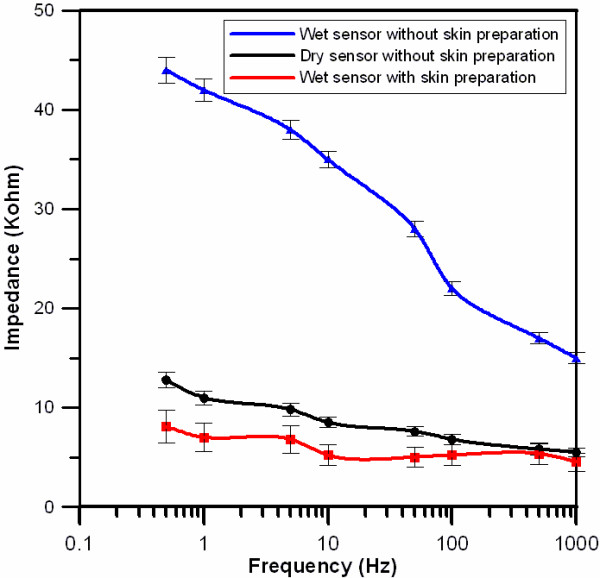
**The skin-sensor contact impedance values on the forehead (F10), with frequencies ranging from 0.5 to 1,000 Hz**. The bar used in this figure indicates the standard deviation.

In addition, the impedance at the sensor-skin contact interfaces was also measured using impedance spectroscopy (LCR4235, Wayne Kerr Electronics Ltd., UK) [1]. The conventional EEG sensors were attached to the skin on the left side of the forehead of the users using their self-adhesive properties. The dry EEG sensors were attached with a disposable 3 M strap and were changed carefully between each measurement to avoid any change in the skin surface [36]. The user's skin was cleaned by gently wiping it with a cotton pad with 2-propanol, which was allowed to evaporate before applying the sensors [1]. To guarantee reliable and reproducible results, the test signal of the impedance spectroscopy was set to 1 V, and the frequency range was set at 0.5 to 1,000 Hz [37,45]. Ten tests were performed on five different participants for the two different EEG sensors (wet and dry). Figure 9 shows the impedance measurements for the different conditions. In Figure 9, the black line indicates the impedance of the dry EEG sensor pair without the skin preparation or conductive gel. The blue and red lines denote the impedances of the conventional EEG sensors, without and with the skin preparation, respectively. All of the conventional EEG sensors were applied with conductive gel. The results indicated that the impedance levels between the skin and the dry EEG sensors without skin preparation or conductive gel were close to that of the conventional wet EEG sensors with skin preparation and conductive gel on the forehead site (F10). Therefore, the dry EEG sensor shown its potential to compare with the conventional EEG sensor in terms of conduction performance [37].

**Figure 10:**
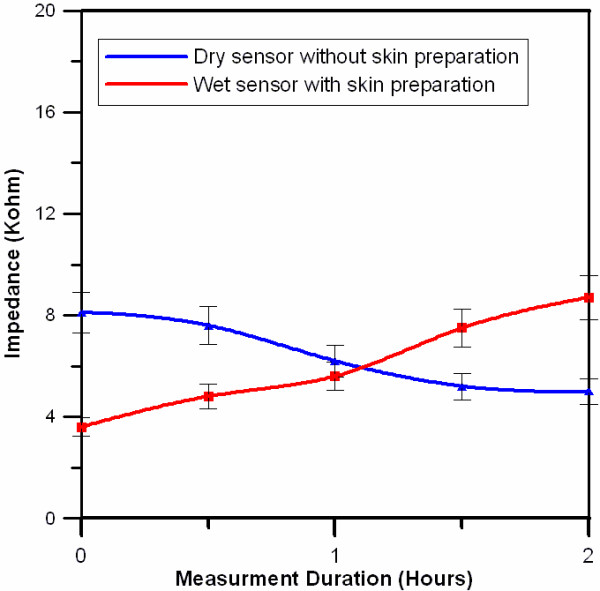
**Comparison of the impedance variation levels between the wet and dry sensors during long-term (2-hour) measurements on the forehead site (F10)**. The bar used in this figure indicates the standard deviation.

Figure 10 shows the impedance variation for the different sensors (wet and dry sensors) during long-term EEG measurements. For the long-term EEG measurements, the impedance variation of the conventional EEG sensor with conductive gel and skin preparation was higher than that of the dry EEG sensor. The impedance variation of the dry EEG sensor was observed to be in the range of 4 to 12 kOhm and was within the acceptable range for normal EEG measurements [37]. Furthermore, compared with the conventional EEG sensor during long-term EEG measurements (2 hours), the dry sensors provided reliable signal quality in terms of skin-electrode impedance (Figure 10) [31]. This result can be explained by the fact that the dry EEG sensor does not require conductive gel, which tends to dry during measurements and therefore reduce stability relative to the wet sensor.

### B. Results of the relationships between short-term memory testing, FF values and BCI gaming scores under quiet and noisy conditions

The accuracies of all trials for the short-term memory experiment were calculated under quiet and noisy conditions, and a paired *t*-test was performed to determine whether they showed any significant differences under the two conditions, as shown in Table 1. The average accuracy levels were 69.0% and 59.8% under the quiet and noisy conditions, respectively. The trends of the results showed a significant decrease in user accuracy under noisy conditions relative to quiet conditions for all users (*p *< 0.05). In addition, the measurement results of the FF values under two different conditions and the corresponding *t*-test data are shown in Table 2. The average FF values were 6.94 and 4.64 under the quiet and noisy conditions, respectively. The average FF values also showed a significant decrease under noisy conditions relative to quiet conditions (*p *< 0.05). Our results are in agreement with the initial assumptions that the users maintained a lower FF under noisy conditions than under quiet conditions because of the presence of distractions [40,42,43]. Moreover, Bobbs *et al*. not only performed the short-term memory experiment to test the users' mental performance, but they also discussed the difference between extroverts and introverts under quiet and noisy conditions [42]. They found that both extroverts and introverts displayed a significantly more focused state under quiet conditions than under noisy conditions. Our data are consistent with those of previous studies [42].

**Table 1:** Results of the short-term memory experiment under quiet and noisy conditions.

	Quiet Condition			Noisy Condition			
		
	Total	Correct	Accuracy	Total	Correct	Accuracy	*p*-value*
**Subject 1**	32	24	0.750	22	14	0.636	
**Subject 2**	44	34	0.773	42	28	0.667	
**Subject 3**	41	25	0.610	47	27	0.574	
**Subject 4**	36	24	0.667	38	21	0.553	
**Subject 5**	58	35	0.603	55	32	0.582	
**Subject 6**	53	38	0.717	51	25	0.490	
**Subject 7**	53	36	0.679	54	31	0.574	
**Subject 8**	54	35	0.648	55	29	0.527	
**Subject 9**	48	35	0.729	46	31	0.674	
**Subject 10**	50	36	0.720	47	33	0.702	

			0.690			0.598	0.001
* Paired ***t***-test.							

**Table 2:** Results of the FF values and gaming scores under quiet and noisy conditions.

	FF			Game Score		
	
	Quiet	Noisy	*p*-value*	Quiet	Noisy	*p*-value*
**Subject 1**	8.0	4.9		9.6	7.4	
**Subject 2**	8.7	4.5		8.4	7.0	
**Subject 3**	5.4	4.4		9.2	7.6	
**Subject 4**	6.1	4.2		9.0	8.2	
**Subject 5**	4.5	3.5		9.0	7.6	
**Subject 6**	8.9	4.1		9.1	6.9	
**Subject 7**	7.1	3.9		9.1	6.1	
**Subject 8**	5.9	4.6		9.1	8.1	
**Subject 9**	8.0	6.6		8.7	7.9	
**Subject 10**	6.8	5.6		9.1	7.1	

	6.940	4.642	0.0005	9.013	7.393	0.00004
* Paired ***t***-test.						

In addition, to ensure the relationship between mental focus and the measured FF values, a Pearson product-moment correlation was performed to determine whether the results of the short-term memory experiment were truly related to the measured FF values [40,46]. According to the Pearson correlation results, indicate that the results of the short-term memory experiment were highly positively related to the measured FF values under both quiet (r = 0.918) and noisy (r = 0.658) conditions [40,46]. According to the results of the Pearson correlation, the measured FF values were significantly positively correlated to the results of the short-term memory experiment. Thus, the measured FF values truly represented the users' level of mental focus. The measured FF values were also used to quantify the gaming scores (FL values).

The total gaming scores under quiet and noisy conditions are shown in Table 2. The average score was 9.01 under quiet conditions and 7.39 under noisy conditions. The game scores showed a significant difference between noisy and quiet conditions (*p *< 0.05). Note that the game scores are positively correlated to the measured FF values. Thus, it is significant that the game scores are lower if the user performs the test under noisy conditions or in the unfocused state. These experimental results show that the FF values are an indicator of the focused state and that the FL algorithm is a reliable method for measuring the users' level of focus via EEG signals. Our results indicate that using the FL algorithm to measure the focus level of the user is useful not only in the context of short-term memory, but also in the measurement of daily life activities. Traditionally, users only perform the short-term memory experiment during testing; these methods are not used to test the users' focused state in other cognitive experiments [39]. Applications of the short-term memory experiment have not been explored in combination with other cognitive testing procedures [41]. However, according to our results, using the novel EEG-based BCI device with the FL algorithm, users can undertake the cognitive experiment without any inconvenience while simultaneously undergoing measurement of the focused state. Moreover, with the proposed device, it is possible to display real-time feedback to remind the users' focus state, which traditional short-term memory tests cannot do. Accordingly, this is a significant advantage of the FL algorithm with our portable EEG-based BCI device, which does not require any skin preparation, over traditional approaches.

## Conclusions

In the present study, we proposed a wearable EEG-based BCI device with dry EEG sensors for cognitive state monitoring. In addition, we demonstrated its use during EEG-based gaming control. The use of dry EEG sensors provides several advantages: 1) in contrast to conventional EEG sensors, the dry foam-based sensors can be used without conductive gel; 2) the elasticity of the substrate of the dry EEG sensors allows them to adapt to irregular skin surfaces to maintain low sensor-skin impedance; and (3) the fabrication process is inexpensive, comparing with other types of dry sensors. Our experimental results demonstrated successful, stable EEG measurements using the dry foam-based EEG sensors through the corresponding wireless EEG acquisition device; these results were almost identical to those seen with conventional EEG sensors in which conductive gel are used. Therefore, in contrast to the conventional EEG-based BCI devices using the wet sensors, our device with dry foam-based EEG sensors have the potential for allowing routine and repetitive measurements. Moreover, a portable, wireless and low-power-consumption EEG acquisition module was successfully used for long-term EEG monitoring. The dry EEG sensors and the wireless EEG acquisition module were embedded into a wearable EEG acquisition device. Using our wearable EEG-based BCI device without conductive gel will allow users to monitor their EEG states more comfortably during daily life.

A cognitive application of EEG-based gaming control was also demonstrated in this study using this portable device. A personal computer was used as the platform to run a real-time focused feature detection algorithm and an EEG monitoring program, which were used to monitor the user's cognitive state. Our data indicate that this wearable EEG-based BCI device and the corresponding algorithm can be reliably used to control outside-world applications for general users or researchers. This device complements other existing BCI approaches for investigating the human cognitive states of neuronal activation and behavioral responses in daily life.

## Competing interests

The authors declare that they have no competing interests.

## Authors' contributions

LDL and CYC conceived of the study design, performed the data collections and processing, and drafted the manuscript. IJW, SFC, SYL and BWC help to develop the wireless readout circuit. JYC and CTL revised the paper for consistency in the neurophysiological aspects. All authors read and approved the final manuscript.
